# F238. COMPETENCE-PERFORMANCE DISCREPANCY IN SOCIAL FUNCTIONING IN PATIENTS WITH SCHIZOPHRENIA: THE IMPACT OF SOCIAL ANXIETY

**DOI:** 10.1093/schbul/sby017.769

**Published:** 2018-04-01

**Authors:** Takahiro Nemoto, Takashi Uchino, Sayaka Aikawa, Tomoyuki Funatogawa, Hiroshi Matsumoto, Taiju Yamaguchi, Naoyuki Katagiri, Naohisa Tsujino, Kei Sakuma, Masafumi Mizuno

**Affiliations:** Toho University School of Medicine

## Abstract

**Background:**

Social functioning deficits are of critical importance in patients with schizophrenia, because they affect the long-term outcomes and quality of life (QOL) of the patients. Two aspects of social functioning, namely, competence (ability to perform skilled activities, that is, what one can do) and performance (actual performance of skilled activities, that is, what one actually does) are considered to have a significant influence on how well the patients can live independently in the community. Although the two aspects are usually thought to go hand in hand, discrepancy between the two is often observed in patients with schizophrenia in clinical practice. Some patients are not able to function in the community to the best of their ability; some patients appear to get along everyday living better than they would be expected to. The aim of the present study was to identify factors influencing the occurrence of such discrepancy of social functioning in patients with schizophrenia.

**Methods:**

A total of 205 stable outpatients with schizophrenia aged 40 years old or under were recruited at the Toho University Omori Medical Center, Tokyo. Of the 205 patients, 100 were male (48.8%) and 105 (51.2%) were female. The mean age of the participants was 29.3 years and the mean estimated premorbid IQ was 100.8. The mean age at disease onset was 22.0 years old, and the mean duration of illness at the start of the study was 6.7 years. The social functioning, psychiatric symptoms, social anxiety, cognitive function, and QOL of the participants were assessed. The patients were divided into 4 groups by the cutoff points for competence and performance calculated using a comprehensive dataset of the Social Functioning Scale (SFS) obtained from multiple facilities.

**Results:**

The subjects were divided according to their level of competence and performance as follows: good competence and good performance (CP) group, 108 (52.7%) patients; good competence but poor performance (Cp) group, 40 (19.5%) patients; poor competence but good performance (cP) group, 13 (6.3%) patients; poor competence and poor performance (cp) group, 44 (21.5%) patients. Among the 4 groups, the objects of particular interest in this study were the differences between CP and Cp groups and between the cP and cp groups. One-way ANOVA revealed significant differences among the groups in the scores on the Positive and Negative Syndrome Scale (PANSS), Liebowitz Social Anxiety Scale (LSAS), Global Assessment of Functioning Scale (GAF), World Health Organization-Quality of Life 26 (WHOQOL26), and Social Functioning Scale (SFS). Post-hoc comparisons revealed that the PANSS negative symptoms and general psychopathology scores, GAF score, WHOQOL26 score, and SFS total score were significantly worse in the Cp group than in the CP group, and that the LSAS score, GAF score, WHOQOL26 score, and SFS total score were significantly better in the cP group than in the cp group.

**Discussion:**

In patients who are capable of living well in the community but do not perform well, negative symptoms may be involved in this discrepancy of social functioning. Patients who are able to maintain themselves well despite their poor social competence appear to have milder social anxiety symptoms as compared to patients who are neither competent nor capable of performing well in terms of social functioning in the community. Suitable and personalized approaches based on the patients’ profile of dysfunction would seem to be indispensable for the recovery of such patients.

